# Stock Portfolio Optimization Using a Combined Approach of Multi Objective Grey Wolf Optimizer and Machine Learning Preselection Methods

**DOI:** 10.1155/2022/5974842

**Published:** 2022-08-29

**Authors:** Nasrin Bagheri Mazraeh, Amir Daneshvar, Mahdi Madanchi zaj, Fereydon Rahnamay Roodposhti

**Affiliations:** ^1^Department of Financial Management, Science and Research Branch, Islamic Azad University, Tehran, Iran; ^2^Department of Information Technology Management, Electronic Branch, Islamic Azad University, Tehran, Iran; ^3^Department of Financial Management, Electronic Branch, Islamic Azad University, Tehran, Iran; ^4^Department of Accounting, Islamic Azad University, Science and Research Branch, Tehran, Iran

## Abstract

The present paper deals with optimizing the stock portfolio of active companies listed on the Tehran Stock Exchange based on the forecast price. This paper is based on a combination of different filtering methods such as optimization of trading rules based on technical analysis (ROC, SMA, EMA, WMA, and MACD at six levels—Very Very Weak (VVW), Very Weak (VW), Weak (W), Strong (S), Very Strong (VS), and Very Very Strong (VVS)), Markov Chains, and Machine Learning (Random Forest and Support Vector Machine) Filter stock exchanges and provide buy signals between 2011 and 2020. In proportion to each combination of filtering methods, a buy signal is issued and based on the mean-variance (M-V) model, the stock portfolio is optimized based on increasing the portfolio return and minimizing the stock portfolio risk. Based on this, out of 480 companies listed on the Tehran Stock Exchange, 85 active companies have been selected and stock portfolio optimization is based on two algorithms, MOGWO and NSGA II. The analysis results show that the use of SVM learning machine leads to minor correlation error than the random forest method. Therefore, this method was used to predict stock prices. Based on the results, it was observed that if the shares of companies are filtered, the risk of transactions decreases, and the return on the stock portfolio increases. Also, if two filtering methods are applied simultaneously, the stock portfolio returns slightly and the risk increases. In the analysis, MOGWO algorithm has obtained 133.13% stock return rate with a risk of 3.346%, while the stock portfolio returns in NSGA II algorithm 107.73, with a risk of 1.459%. Comparison of solution methods shows that the MOGWO algorithm has high efficiency in stock portfolio optimization.

## 1. Introduction

Investment is defined as the investment of money to obtain additional or certain benefits relative to money. In addition to providing benefits (returns), the investment also has a risk borne by the investor. The higher the rate of return expected by an investor, the higher the risk to be covered by the investor [1]. The level of risk can be minimized at a certain rate of stock portfolio expectations by forming an appropriate portfolio. Therefore, stock portfolio optimization has an important role in determining investment portfolio strategies for investors. What investors hope to achieve through portfolio optimization is to maximize stock portfolio returns and minimize stock portfolio risk. Because returns vary based on risk, investors need to balance the contrast between risk and return on their investment. Therefore, there is no optimal basket that can satisfy all investors. The optimal portfolio is determined by the risk preferences and return of the investor [2].

Based on this, many researchers have analyzed financial data and portfolio optimization because the financial market is full of uncertainty. For example, because of government policies, the global economy, social activities, psychology, and many assets such as stocks, futures, and options, the nonlinear nature and dynamics with high internal dependence have led to the challenge of researchers [3, 4]. The extreme sensitivity of the stock market to political and economic changes and the unstable and nonlinear properties of financial time series have made market forecasting challenging [5–7]. Also, drastic changes in prices and markets at different times require more attention and quick reactions to these changes [8].

Given the importance of investing in portfolio optimization, in 1952, Markowitz proposed a popular model called the mean-variance (M-V) model for stock portfolio weight [9, 10]. Given the expected value at risk (VaR), the M-V model can be used to find the best portfolio that can maximize return on investment (ROI) and reduce their risks. Due to the large number of listed companies, it is impossible to optimize the stock portfolio with normal methods and there is a possibility of increasing the stock portfolio risk. Due to severe market fluctuations, new research on portfolio optimization in recent years has used the H&B method to optimize its portfolio [11]. In this method, regardless of market fluctuations, shares of listed companies are purchased and held until the end of the investment period. Although this method reduces the risk of investing, there is no expectation of high return on investment.

Researchers, considering the higher investment risk used different methods to receive the buy, hold, or sell signal. These methods include machine learning models, time series, Markov chains, meta-heuristic algorithms, and so on. Different models of stock price forecasting, considering the high computational ability, offer the best combination of stock portfolio optimization by considering lower risks [12]. In these methods, stock transactions are reviewed based on existing theories and based on the training provided, the best time to enter or exit the market is presented. As a result, the risk of the transaction refers to the risk arising from the time of the transaction. This is the time due to an error in estimating future price movements, which causes the trader to place his or her order at the desired price based on his or her predictions, and faces the risk of not fulfilling the order at the desired price. In this case, it has to trade at a more unfavorable price than the market price at the time of ordering [13]. Therefore, there is always an exchange between cost and ordering risk in executing an order. This is an issue that has been described as a trade-off [14]. Therefore, balancing the market reaction cost with the opportunity cost risk requires designing trading strategies. In choosing a trading strategy, the investor, in addition to adjusting his or her preferences, must be prepared to change and adapt the strategy to changing market conditions. The task of designing an optimal transaction strategy is complex because it requires decisions about the best way to split the order, the investor's entry point in order execution, the choice of order type, and how to measure transaction performance. In the past, the task of designing a trading strategy was usually the responsibility of experts (humans), but with the increase in the number of decisions, an automated approach is needed. Therefore, the method must be dynamic and responsive to market conditions in real time [15].

According to market theory, investors cannot predict stock prices and market conditions based on past information [16]; therefore, they should quickly identify the information that affects the stock price and adjust their prices according to the information. It is impossible to control a large number of stocks at the same time. Therefore, market information must be transmitted through various filter processes and signals of purchase, sale, or maintenance [17–19]. Efficient market theory does not rule out the possibility of repetitive and short-term patterns. If these hidden patterns can be identified before they occur, the market's future can be accurately predicted. Many projects have been undertaken to forecast financial markets. Some of these studies have used soft computing techniques such as genetic algorithms, neural networks, and fuzzy systems. Researchers therefore use sophisticated methods to reduce prediction error. Price forecasting in financial markets is an attractive topic of interest for market participants, both personal and institutional investors. Investment in today's world faces major challenges that the methods of selecting and forming the appropriate portfolio algorithmically can address these challenges.

In this paper, due to the importance of considering risk in investment, stock portfolio optimization with the aim of increasing eleven portfolios and reducing the risk of investing in the portfolio simultaneously on the shares of companies listed on the Tehran Stock Exchange is discussed. According to the information contained in the information site of the Tehran Stock Exchange Technology Management Company (TSETMC), there are 480 companies listed on the Tehran Stock Exchange between 2011 and 2020. Therefore, in this article, we first filter the active companies based on three categories of input information such as 8 technical variables (final price, number of buyers, number of transactions, trading volume, daily trading value, company day value, *P*/*E* ratio, and number of shares Per company), 7 fundamental variables (old design coin price, new design coin, dollar, index, gold, oil, euro), And the time series of the final price of the last 10 days of each share is considered and companies that have traded at least one day per month. The importance of market price forecasting has also led to different ways of filtering and presenting buy signals such as filtering “Optimization of trading rules based on technical analysis (Trading Rule Filter),” “Optimization of a strong trading system using Markov chain (Markov Model Filter),” and “Optimize and predict stock returns based on Predict Model Filter methods.” In Trading Rule Filter, 6 indicators RSI, ROC, SMA, EMA, WMA, and MACD in six levels, very very weak (VVW), Very weak (VW), Weak (W), Strong (S), Very Strong (VS), and very very strong (VVS) has been used. In the Predict Model Filter of data training, machine learning methods (random forest method and processing vector machine) are used. After determining the buy signal, the MOGWO and NSGA II algorithms for stock portfolio optimization based on the M-V model are discussed and the average stock portfolio return as well as the average stock portfolio risk are examined and compared.

Based on the presented issue, the innovations of the paper can be summarized as follows:

- Using three filters for buy signal such as “Optimization of trading rules based on technical analysis (Trading Rule Filter),” “Optimization of a strong trading system using Markov chain (Markov Model Filter),” and “Optimize and predict stock returns based on Predict Model Filter methods”
- Using M-V model to portfolio optimizing
- Using MOGWO and NSGA II algorithms to optimization M-V model

In the M-V model presented in this research, it is possible to simultaneously maximize stock portfolio profit and minimize investment risk, and the use of Meta heuristics algorithms leads to the optimization of the objective functions of the M-V model and the achievement of a various investment set.

The structure of the paper is as follows. In the second part, the background of research related to stock portfolio optimization and stock portfolio risk is discussed. In the third part, a multi-objective stock portfolio selection and optimization model based on technical process, Markov decision-making and learning machine is presented, and a two-objective model for stock portfolio optimization is presented. In the fourth section, the results of the implementation of the approach on the data of listed companies operating in Iran are analyzed and presented. In the fifth section, conclusions and future recommendations of the research are presented.

## 2. Literature Review

Research conducted in foreign markets using a combination of different strategies to support foreign market optimization algorithms shows promising results. On the other hand, there are different approaches to evolutionary computing strategies, and machine learning algorithms, which include neural networks, genetic algorithms, and support vector machine algorithms. In research on financial market forecasting of artificial intelligence algorithms including artificial neural networks [20–23], recursive neural networks [24], genetic algorithms with neural networks, artificial [25, 26] and support vector neural networks [27] have been used. Quek et al. [28] proposed the use of fuzzy neural network (GenSoFNN) as a tool for stock portfolio balancing, which uses a supervised learning method to detect milestones in the stock price cycle and a modified weighted regression algorithm used to smooth the stock cycle. Hsu et al. [29] used Markov chains and fuzzy theory to create a stock market index forecasting model. Following the validation of the data, the results show not only the ability to improve the return on investment but also the prevention of losses. Chiu and Chian [30] designed new functional genetic algorithms to select investment targets based on stock price regardless of whether the stock market index is declining. This method can achieve sustainable returns. Leu and Chiu [31] described integrated genetic algorithms and pattern search methods for capital allocation and stock selection. Such experiments with the usual selection methods lead to good results with the roulette wheel method. Dastkhan et al. [32] used fuzzy time series to predict return on investment and then genetic algorithms to identify the optimal investment strategy. Experimental results showed this method for the performance of 60 Taiwanese market indices. Sefiane and Benbouziane [33] in a paper entitled Portfolio Selection Using Genetic Algorithm used the genetic algorithm on a simple example containing 6 shares. They note that the results are interesting and confirm the efficiency of the genetic algorithm for its rapid convergence to a better solution, as well as the computational time. Van de Vrande [34] by examining the effects of portfolio diversity on performance results and the conditions in which diversity is likely to occur and using a data set of strategic investments by pharmaceutical companies showed that the variance is in the relative technology proximity between the central company. And, its partners show the inverted relationship of U-shape with innovative performance, which is influenced by the diversity of external source modes used in the portfolio. Abounoori et al. [35] in a study entitled Predicting Fluctuations in the Tehran Stock Exchange with Markov GARCH approach evaluated several GARCH models in relation to their ability to predict fluctuations in the Tehran Stock Exchange. Chen and Hao [36] provide a technical analysis of a set of technical characteristics such as moving average and exponential moving average and convergence-divergence mean motion and volume ratio and relative power index and volume level and acceleration index for prediction with learning algorithms. Basak et al. [37] presented a stock market forecast using random forest. An empirical classification framework for stock price prediction, given the previous day's price increase or decrease, facilitates this relationship by using random forests and decision trees. Molchanov and Romasheva [38]. In order to achieve the goals and maximize profitability, companies suggested that indicators should be created for a balanced portfolio, which would allow portfolios to be evaluated and current and potential projects ranked to create flexibility with minimal risk. They analyzed modern approaches and benchmarking firms for portfolio management, current industry situation, identified risks, and indicators for evaluation. Chang and Young [39] in a study investigated the optimization of the stock portfolio by exploiting the cause-and-effect relationships of return and bias in behavioral stocks according to the return of the behavioral stock retention period. By mimicking real investment constraints, including test costs and statistical evidence, they found that by including short selling in stock portfolio selection, the investment flexibility available and the stock portfolio outperformed the benchmarks and the market. Jiang et al. [40] proposed a stock index forecasting model using deep learning algorithms. This input model of technical indicators along with macroeconomic indicators was used to forecast the stock price index on a monthly basis. Chowdhury et al. [41] presented stock price forecasting using the Black Scholes pricing model and machine learning. Different algorithms such as decision tree, machine learning method, and neural network have been used. Pungetmongkol et al. [44] examined the performance of a balanced portfolio and a shopping cart for five different market trends: uptrends, downtrends, and market sideways. They considered three asset classes, namely, equity, bonds, and gold, determined the initial weight of the portfolio using the Markowitz method, and used geometric Brownian motion to simulate prices.

Bigerna et al. [43], considering the amount of oil imports and the risk associated with this amount, introduced a new approach to the concept of energy security, which from the perspective of the portfolio theory to examine these issues, from the perspective of four major Asian energy importers; Used by China, Japan, Korea and Taiwan. Wang et al. [44] proposed a reinforcement learning method for optimizing investment policy. To address the problem of balance of risk and return, they proposed a model that uses macro market conditions as a dynamic indicator to adjust the ratio between short and long funds to reduce the risk of market fluctuations. Óskarsson [45] conducted a study aimed at retesting theories and analyzing the impact of risk management tools on different collections, focusing on investing in Iceland, where stock price fluctuations and foreign exchange rates can change portfolio returns over time. And affect investor profitability. dos Santos et al. [46] conducted a study to identify which tools and techniques are more appropriate for the strategic selection and alignment of projects and the balance of the project portfolio. Papavasiliou and Bertrand [47] in a study aimed at developing a methodology that demonstrates the consequences of a balanced European market that undergoes a fundamental transformation through multiple market design schemes. Their method relies on analytical insights that can be obtained under the assumption of pricing behavior. They used a simulation model that demonstrates the European equilibrium market as a Markov decision-making process to confirm this. Hernandez-Vega [48] studied how the unexpected announcements of US monetary policy affect the inflow of foreign investment. They used a new set of data on debt and net daily flows. The results showed that both equity and liabilities reacted immediately to announcements, especially bad news. Sawik [49] proposed a model with the aim of determining the optimal investment in cyber security under a limited budget and a set of security controls for each supply chain node to balance cyber security throughout the supply chain. Using lattice conversion, together with Taylor's first series estimation of natural logarithm, they approximated a nonlinear stochastic hybrid optimization model with its linear equivalent. Some authors have used meta-heuristic algorithms to optimize the portfolio, such as the HI algorithm [50], WOA algorithm [51, 52], ICA-FA algorithm [53], GWO algorithm [54, 55], and the IWO algorithm [56].

According to the research background, it can be said that different methods have been used to optimize the stock portfolio and reduce the risk of the investment portfolio. However, there is no comprehensive model that uses both learning machines (random warfare and processing vector machines) and the Markov decision-making process for training and NSGA II and MOGWO algorithms for data testing.

## 3. Problem Definition and Solving Method

**Figure 1:**
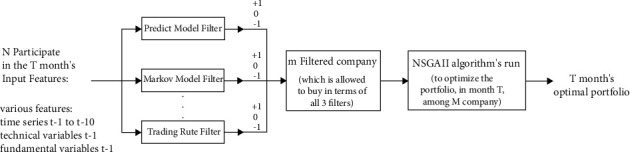
Proposed model general market timing.

In this section, the paper optimizes the stock portfolio of stock companies based on the Markowitz mean-variance model. The main purpose of this section is to provide a model to increase the rate of return on the stock portfolio and reduce investment risk. For this purpose, the companies listed on the stock exchange have been studied. Since investing in all stocks of listed companies is impossible and the risk of investing in them is high, in this section, some of the listed companies are first filtered using predefined rules. The reason for this is to focus on companies that can be traded at least once a month. In order to filter active companies from other companies, three types of input data are used according to Figure 1:

1. Eight technical variables (final price, number of buyers, number of transactions, trading volume, value of daily transactions, daily value of the company, *P/E* ratio, and number of shares of each company)
2. Fundamental variables (old design coin price, new design coin, dollar, index, gold, oil, euro)

## 4. Time Series of the Final Price of the Last 10 Days of the Share per Month

The existence of different fundamental variables leads to an impact on stock prices, and therefore these variables are very effective in determining the purchase, maintenance, or sale of shares. Due to the existence of two different types of coins in Iran with different prices, in this section the term old and new design has been used.

According to the three decision variables, the following techniques have been used to filter the studied shares of stock exchange companies based on the buying and selling signals of low-yielding companies:

- Optimization of trading rules based on technical analysis (Trading Rule Filter)
- Optimize a robust trading system using the Markov Model Filter
- Optimization and prediction of stock returns based on machine learning methods (Predict Model Filter)

The following is a description of each of the filters based on receiving a buying signal and eliminating low-yield companies:

### 4.1. Technical

**Figure 2:**
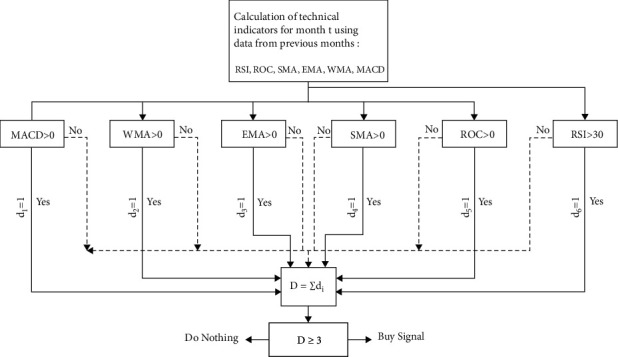
Performance of the technical rule to provide a buying signal.

Technical analysis is a business discipline that is used to evaluate investments and identify trading opportunities by analyzing the statistical trends collected from trading activities, such as price and volume movements. Technical analysis often uses a variety of chart tools to generate short-term trading signals, and it can also help improve the valuation of the strength or weakness of a security over a wider market or segment. This information helps analysts improve their overall valuation estimate. In technical methods, disabilities can be considered as signposts for a route. This paper out of 6 indicators RSI, ROC, SMA, EMA, WMA and MACD in six levels Very Very Poor (VVW), Very Poor (VW), Weak (W), Strong (S), Very Strong (VS) and Very Very Strong (VVS) is used to filter stock companies. According to Figure 2, if at least 3 of the indicators issue a buying signal, that share of the company will be considered for the next day's purchase.

### 4.2. Markov Chain

A Markov process is a stochastic process that satisfies the Markov property (sometimes characterized as “memorylessness”). In simpler terms, it is a process for which predictions can be made regarding future outcomes based solely on its present state and—most importantly—such predictions are just as good as the ones that could be made knowing the process's full history. In other words, conditional on the present state of the system, its future and past states are independent. A Markov chain is a type of Markov process that has either a discrete state space or a discrete index set (often representing time), but the precise definition of a Markov chain varies. For example, it is common to define a Markov chain as a Markov process in either discrete or continuous time with a countable state space (thus regardless of the nature of time), but it is also common to define a Markov chain as having discrete time in either countable or continuous state space (thus regardless of the state space).

The system's state space and time parameter index need to be specified.  Table 1 gives an overview of the different instances of Markov processes for different levels of state space generality and for discrete time *v*. continuous time.

Note that there is no definitive agreement in the literature on the use of some of the terms that signify special cases of Markov processes. Usually the term “Markov chain” is reserved for a process with a discrete set of times, that is, a discrete-time Markov chain (DTMC), but a few authors use the term “Markov process” to refer to a continuous-time Markov chain (CTMC) without explicit mention. In addition, there are other extensions of Markov processes that are referred to as such but do not necessarily fall within any of these four categories. Moreover, the time index need not necessarily be real-valued; like with the state space, there are conceivable processes that move through index sets with other mathematical constructs. Notice that the general state space continuous-time Markov chain is general to such a degree that it has no designated term.

While the time parameter is usually discrete, the state space of a Markov chain does not have any generally agreed-on restrictions: the term may refer to a process on an arbitrary state space. However, many applications of Markov chains employ finite or countably infinite state spaces, which have a more straightforward statistical analysis. Besides time-index and state-space parameters, there are many other variations, extensions, and generalizations. For simplicity, most of this article concentrates on the discrete-time, discrete state-space case, unless mentioned otherwise.

According to the contract, we assume that there is always the next case and as a result the process continues forever. According to this method, groups of data similar to today's data are examined based on the time series of the data in question and the amount of increase or decrease of the average price is calculated. Therefore, based on the current day price and the forecast for the next day price, a buy signal will be issued.

### 4.3. Machine Learning

Machine learning is the scientific study of statistical algorithms and models used by computer systems that use patterns and inference to perform tasks instead of using explicit instructions. It is a science that allows computers to learn a particular subject without the need for an explicit program. As a subset of artificial intelligence, machine learning algorithms create a mathematical model based on sample data or “training data” for predicting or making decisions without obvious planning. The goal of machine learning is for computers and systems to be able to perform their tasks gradually and with increasing data. The scope of this task can range from automatic face recognition by seeing a few examples of the desired face to learning how bipedal robots walk by receiving a stock buy signal. The range of research that can be done on machine learning is wide. Theoretically, researchers believe that they should create new learning methods and study the feasibility and quality of learning for their methods, and on the other hand, some researchers try to apply machine learning methods to new problems. Of course, this spectrum is not discrete and the researches have components of both approaches. Machine learning helps a lot in saving operating costs and improving the speed of data analysis.

In this paper, two different types of machine learning methods based on support vector machine (SVM) and random forest (FR) are used. The machine learning method, in addition to filtering company stocks, also issues a buy/hold/sell signal for the next day. Also, in the vector machine processing method, 20 similar basic learning models have been used to reduce the prediction error. In the following, each of the mentioned methods is described.

### 4.4. Random Forest

**Figure 3:**
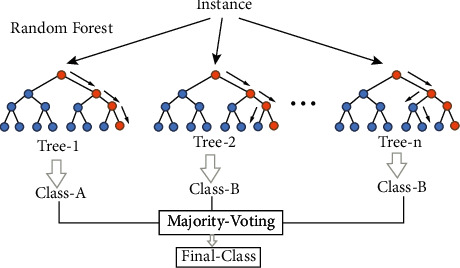
Random forest performance.

Random forest or random forests (RF) is a combination learning method for classification, regression, which is based on a structure consisting of a large number of decision trees, on the training time and output of classes (classification) or for each tree predictions. They work in a separate form. Random forests are suitable for decision trees that are overfitted in the training complex. Random forest performance is usually better than the decision tree, but this performance improvement depends in part on the type of data. Stochastic forest is a supervised learning algorithm. As the name implies, this algorithm builds a forest randomly. The “forest” created is actually a group of “Decision Trees.” The construction of the forest using trees is often done by the method of “bagging.” The main idea of the bagging method is that a combination of learning models enhances the overall results of the model. Simply put, a random forest decides several trees and merges them together to make more accurate and consistent predictions. One of the advantages of random forest is that it can be used for both classification and regression problems, which make up the majority of current machine learning systems. Here, the function of random forest for “classification” will be described, as classification is sometimes considered the building block of machine learning. Random forest adds added randomness to the model as trees grow. Instead of searching for the most important properties when splitting a “node,” the algorithm looks for the best properties among a random set of properties. This leads to a lot of variety and ultimately a better model. Thus, in a random forest, only one subset of features is considered by the algorithm to split a node. By increasing the random threshold for each attribute instead of searching for the best possible threshold, trees can be made even more random.  Figure 3 shows the random forest method made of a tree.

### 4.5. Support Vector Machine

Support vector machine (SVM) is one of the supervised learning methods used for classification and regression. This method is one of the relatively new methods that have shown good performance in recent years compared to older methods for classification. The basis of SVM classifier work is linear classification of data, and in linear segmentation of data we try to choose the line that has the most reliable margin. Solving the optimal line finding equation for data is done by QP methods, which are known methods for solving constrained problems. One of the methods that are currently widely used for the classification problem is the support vector machine (SVM) method. Perhaps, the current popularity of the support vector machine method can be compared to the popularity of neural networks over the past decade. The reason for this is the ability to use this method to solve various problems, while methods such as the decision tree cannot be easily used in various problems. The vector machine often uses binary classification. It is assumed that there is an *L* observation that each observation consists of pairs in which there is an input vector and a two-state value (1− or +1). The idea of a backup vector machine tries to draw hyperpages in space that optimally differentiate samples of different data classes.

In this paper, after using a combination of the above methods to filter the stocks of listed companies, the Markowitz mean-variance model is used to optimize the stock portfolio. In this model, from the accepted *N* companies, *m* companies are selected to optimize the stock portfolio. The main goal is to determine the percentage of investment in each company (*W*_*i*_, *i* ∈ *m*). To determine the exact amount of investment, two objective functions are considered in accordance with equations (1) and (2). In relation (1), the goal of maximizing the stock portfolio based on the projected profit (*R*_*i*_^*t*(predict)^, *i* ∈ *m*) and in relation (2) the goal of minimizing investment risk or in other words minimizing the covariance between the two shares of the company (cov_*ij*_, *i*, *j* ∈ *m*). The investment must be made in such a way that in accordance with equation (3) the total volume of transactions does not exceed 1.

(1) $$\begin{array}{c} \max\mu p=\;\sum_{i=1}^{m}R_{i}^{t\left(\text{predict}\right)}.W_{i}, \end{array}$$

(2) $$\begin{array}{c} \min\text{Risk}=\sum_{i=1}^{m}\sum_{j=1}^{m}{\mathrm{cov}}_{ij}W_{i}W_{j}\;, \end{array}$$

s.t.:

(3) $$\begin{array}{c} \sum_{i=1}^{m}W_{i}=1, \end{array}$$

(4) $$\begin{array}{c} W_{i}\geq 0. \end{array}$$

According to the Markowitz model, NSGA II and MOGWO algorithms have been used to optimize the stock portfolio based on the two mentioned objective functions. In the following, the mentioned solution methods are presented.

### 4.6. NSGA II Algorithm

One of the most efficient and well-known multi-objective optimization algorithms is the NonDefeatable Genetic Algorithm II (NSGA II). This algorithm is one of the fastest and most powerful optimization algorithms that have less operational complexity than other methods. This algorithm comes to the conclusion that it has an optimal range in terms of changing the objective functions and gives the designer the freedom to choose her desired design from among the optimal designs. In NSGA II, the preservation of elitism and dispersion is considered simultaneously. The selection of a new population in each step of this method is based on the principle of dominance, and using elitism and population ranking in each step of the solution, the best undefeated answers are selected and they go to the next step.

**Figure 4:**
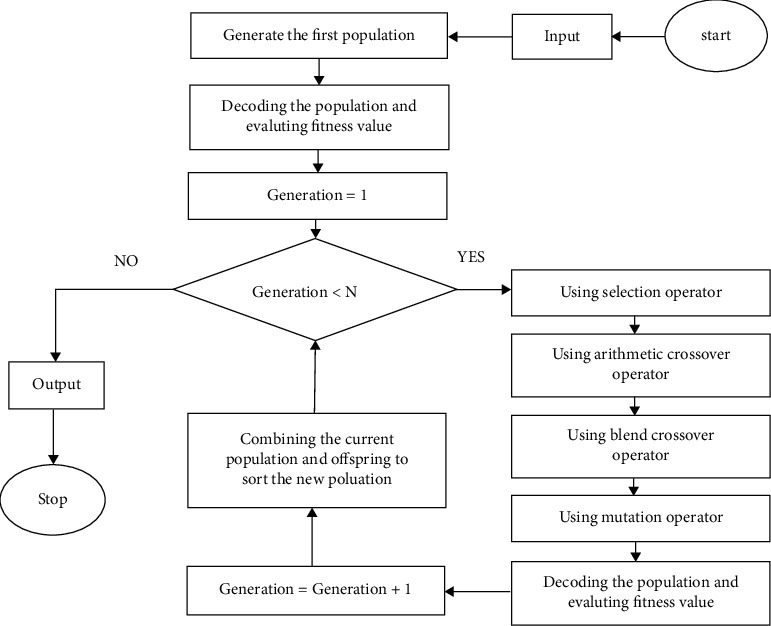
NSGA II algorithm pseudocode in stock portfolio optimization.

If there are two maximization objective functions *f*_1_ and *f*_2_, then for the two answers *x* and *y*, the answer *x* beats the answer *y* if we have *f*_1_(*x*) ≥ *f*_1_(*y*) and *f*_2_(*x*) ≥ *f*_2_(*y*) or *f*_1_(*x*) > *f*_1_(*y*) and *f*_2_(*x*) > *f*_2_(*y*). Also, in order to observe the proper distribution of the density of the answers in this algorithm, a concept called congestion distance is used. In general, to sort a population of size *n* based on nondefeat levels, each answer is compared to all the other answers in the population to determine if that answer is defeated. Finally, there are a number of solutions, neither of which overcomes each other, so these solutions form the first boundary of the invincible boundary. These answers are passed to set *F*_1_. To determine the answers in the next boundaries, the answers in the first boundary are temporarily ignored and the above process is repeated; this time the answers are transferred to the *F*_2_ set and take second place. This process continues for all the unanswered answers of the population. One of the criteria of the evolutionary algorithm in order to reach the optimal Pareto boundary is to maintain the variety and breadth of the answers in the set of obtained answers. In fact, arranging nondefeats is a procedure in order to achieve better answers and the mechanism of diversity also seeks to maintain diversity and breadth in these answers. In this algorithm, this is done by swarming distance in this way. The smaller the swarming distance of an answer, the greater the density of answers around it. For the next step, select the answers that are in the area with less density or in other words with more congestion distance. This increases the diversity and dispersion of the obtained answers. The purpose of using congestion spacing in NSGA II is to create diversity in population responses and to indicate the density of responses alongside a specific response.  Figure 4 also shows the pseudo code of NSGA II algorithm in stock portfolio optimization. The swarm interval for the ordered answers is ascending and specific to set *F* is obtained from.

(5) $$\begin{array}{c} CD\left(X^{1}\right)=CD\left(X^{S}\right)=\infty ,\\ CD\left(X^{i}\right)=\left[\frac{Z_{1}\left(X^{i+1}\right)-Z_{1}\left(X^{i-1}\right)}{Z_{1}\left(X^{S}\right)-Z_{1}\left(X^{1}\right)}\right]+\left[\frac{Z_{2}\left(X^{i+1}\right)-Z_{2}\left(X^{i-1}\right)}{Z_{2}\left(X^{S}\right)-Z_{2}\left(X^{1}\right)}\right],i=2,\ldots ,S-1. \end{array}$$

In the above relation, *CD*(*X*^*i*^) is the amount of congestion distance for the answer *X*^*i*^. After merging the parent-child population, the undefeated sorting is performed and Steps 7 and 8. Based on Step 10, the swarm distance criterion is used to create a subset of the last undefeated set due to the subsequent increase in population size:

**Step 1 .**

Create the initial population *P*_0_ of size *N* with random answers and set *t* = 0,

**Step 2 .**

If the stop condition is not met, return to *P*_*t*_,

**Step 3 .**

Using the binary selection operator, select *N* parents from the population *P*_*t*_,

**Step 4 .**

By applying the intersection and mutation operators on the population *P*_*t*_, generate the population of *Q*_*t*_ children to size *N*,

**Step 5 .**

Set *R*_*t*_=*P*_*t*_ ∪ *Q*_*t*_,

**Step 6 .**

Use the invincible ranking method to determine the Pareto *F*_*i*_ sets in the *R*_*t*_ population,

**Step 7 .**

Set *P*_*t*+1_=∅ و *i*=1,

**Step 8 .**

Until |*P*_*t*+1_|+|*F*_*i*_| < *N*:

- Add the answers of the set *F*_*i*_ to the population *P*_*t*+1_, and
- Put *i*=*i*+1.

**Step 9 .**

Arrange the *F*_*i*_ set answers in descending order of congestion,

**Step 10 .**

Size *N* − *|P*_*t*+1_*|* Transfer from the first answers *F*_*i*_ to the population *P*_*t*+1_, and

**Step 11 .**

Set *t*=*t*+1 and return to Step 2.

### 4.7. MOGWO Algorithm

**Figure 5:**
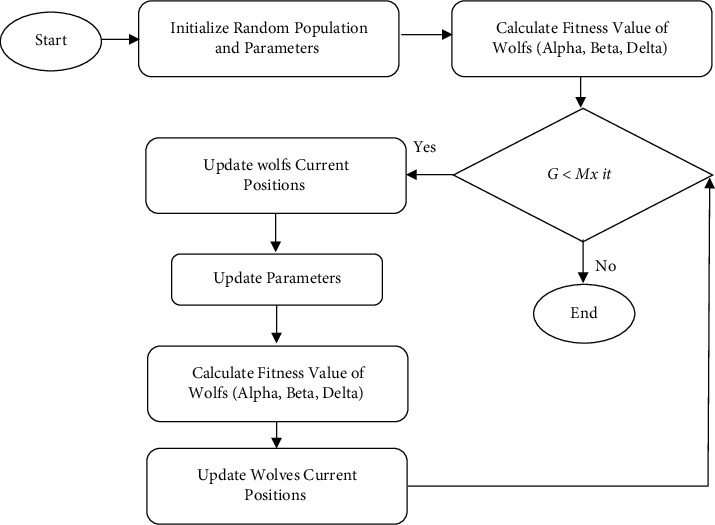
MOGWO algorithm pseudo-code in stock portfolio optimization.

The MOGWO like the NSGA II is a population-based algorithm based on the behavior of gray wolves. In this method, each wolf is introduced as an initial solution (determine the exact amount of investment) which aims to achieve the initial solution that has the highest expected profit and the lowest investment risk. From the MOGWO algorithm based on the equations discussed below; it changes the random response generated in each iteration, which improves the objective functions. Finally, the best initial solution is known as Alpha Wolf, and the result is the highest expected profit and the lowest investment risk. The gray wolf Canis Iupus belongs to the Candidate family. Gray wolves are predators at the top of the food pyramid, meaning they are at the top of the food chain. Gray wolves mostly prefer to live in groups. The average group size is 5–12 wolves. Leaders consist of a male and a female called Alpha. Alpha is primarily responsible for decisions about hunting, where to sleep, when to wake up, and so on. Alpha decisions are communicated to the group; however, some democratic behaviors have also been observed in which an Alpha follows the other wolves in the group. In communities, the whole herd endorses Alpha. Alpha wolf is also known as the dominant wolf, because the commands must be executed by the group. Alpha wolves are only allowed to mate in the herd. It is important to note that Alpha is not necessarily the strongest member of the herd, but the best member in terms of management in the herd. The second level in the gray wolf hierarchy is Beta. Beta are wolves that help Alpha make decisions or other herd decisions. The Beta wolf can be male or female, and he or she is the best replacement for Alpha in the event of his death or aging. Beta executes Alpha commands across the herd and gives feedback to Alpha. The Omega wolf is the lowest class in the gray wolf hierarchy. The Omega Wolf plays the victim. Omega wolves usually have to follow all the high-level and dominant wolves. They are the last wolves allowed to eat. If the wolf is not an Alpha or Omega, it is called a Delta. Delta wolves must be subject to Alpha and Beta. However, they dominate Omega.  Figure 5 also shows the pseudo code of MOGWO algorithm in stock portfolio optimization. In mathematical modeling of wolf social hierarchy, (*α*) Alpha is considered as the most appropriate solution. Subsequently (*β*) Beta and (*δ*) Delta are the second and third most suitable solutions, respectively. The rest of the candidate solutions are assumed to be Omega (*X*). To hunt, gray wolves must find and surround their prey. Therefore, the following equations update the wolves' positions around the prey.

(6) $$\begin{array}{c} \overset{⟶}{D}=\left|\overset{⟶}{C}.\overset{⟶}{X_{p}}\left(t\right)-\overset{⟶}{X}\left(t\right)\right|, \end{array}$$

(7) $$\begin{array}{c} \overset{⟶}{X}\left(t+1\right)=\overset{⟶}{X}\left(t\right)-\overset{⟶}{A}.\overset{⟶}{D}. \end{array}$$

In the above relations $\overset{⟶}{C}$ and $\overset{⟶}{A}$ are coefficient vectors. $\overset{⟶}{X_{p}}$ is the hunting position vector and $\overset{⟶}{X}$ is the gray wolf position vector. This is an equilibrium equation between siege and hunting. Therefore, the search radius must be optimized during the process. For this purpose, the equations related to the two coefficients used in the above relations are as follows.

(8) $$\begin{array}{c} \overset{⟶}{A}=2\overset{⟶}{a}.\overset{⟶}{r_{1}}-\overset{⟶}{a}, \end{array}$$

(9) $$\begin{array}{c} \overset{⟶}{C}=2\overset{⟶}{r_{2}}. \end{array}$$

The above equations enable the gray wolves to update their position around the prey. As a result, the following equations are used to hunt.

(10) $$\begin{array}{c} {\overset{⟶}{D}}_{\alpha }=\left|{\overset{⟶}{C}}_{1}.{\overset{⟶}{X}}_{\alpha }-\overset{⟶}{X}\right|,{\overset{⟶}{D}}_{\beta }=\left|{\overset{⟶}{C}}_{2}.{\overset{⟶}{X}}_{\beta }-\overset{⟶}{X}\right|,{\overset{⟶}{D}}_{\delta }=\left|{\overset{⟶}{C}}_{1}.{\overset{⟶}{X}}_{\delta }-\overset{⟶}{X}\right|, \end{array}$$

(11) $$\begin{array}{c} {\overset{⟶}{X}}_{1}={\overset{⟶}{X}}_{\alpha }-{\overset{⟶}{A}}_{1}.{\overset{⟶}{D}}_{\alpha },{\overset{⟶}{X}}_{2}={\overset{⟶}{X}}_{\beta }-{\overset{⟶}{A}}_{2}.{\overset{⟶}{D}}_{\beta },{\overset{⟶}{X}}_{3}={\overset{⟶}{X}}_{\delta }-{\overset{⟶}{A}}_{3}.{\overset{⟶}{D}}_{\delta }, \end{array}$$

(12) $$\begin{array}{c} \overset{⟶}{X}\left(t+1\right)=\frac{{\overset{⟶}{X}}_{1}+{\overset{⟶}{X}}_{2}+{\overset{⟶}{X}}_{3}}{3}. \end{array}$$

## 5. Analysis of Results

**Figure 6:**
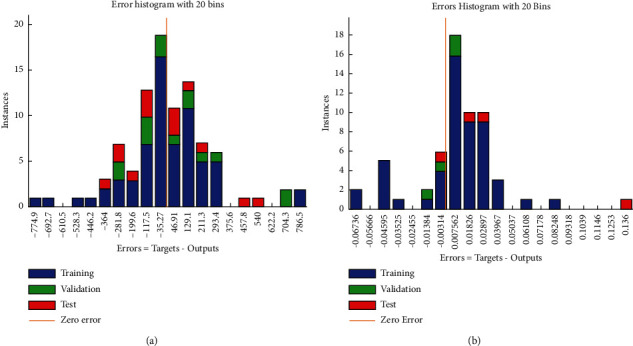
Comparison histogram error for training, evaluation, and testing of data between different machine learning methods. (a) FR method. (b) SVM method.

After presenting different solution methods, in this section, the results of data analysis of companies listed on the Tehran Stock Exchange are reviewed. Based on this, data analysis is done in two stages. In the first stage, companies active in the Tehran Stock Exchange are selected, and in the next stage, after training the data, the return of the portfolio is reduced and the risk of the portfolio is reduced. According to the information extracted from the information site of Tehran Stock Exchange Technology Management Company (TSETMC), 480 companies listed on the Tehran Stock Exchange between 2011 and 2020 have been extracted along with all the stock information of the companies and filtered based on various inputs. To filter and select active stock exchange companies from 2011 to 2020, eight technical variables (final price, number of buyers, number of trades, trading volume, daily trading value, company daily value, *P*/*E* ratio, and number of shares of each company), seven fundamental variables (price of old design coin, new design coin, dollar, index, gold, oil, and euro), and time series of the final price of the last 10 days per share are considered and companies that have traded at least one day a month have been selected. Based on this, 85 companies active in the Tehran Stock Exchange have been identified and selected. After selecting 85 companies active in the Tehran Stock Exchange, to receive a buy signal from different combinations of three types of filters: “Optimization of trading rules based on technical analysis (Trading Rule Filter),” “Optimization of a strong trading system using Markov chain” (Markov Model Filter), and “Optimizing and Predicting Stock Returns Based on Predict Model Filter Methods.” In Trading Rule Filter, 6 indicators RSI, ROC, SMA, EMA, WMA, and MACD in six levels, namely, Very Very Poor (VVW), Very Poor (VW), Weak (W), Strong (S), Very Strong (VS), and very very strong (VVS) have been used. Finally, in the Predict Model Filter data training, machine learning methods are used between 2011 and 2015. In this study, the threshold value of determining the ascending and descending trend is 2% profit and 2% loss, respectively.  Figure 6 shows the machine learning error based on 3 sets of input data based on random forest method (FR) and support vector machine (SVM).

**Figure 7:**
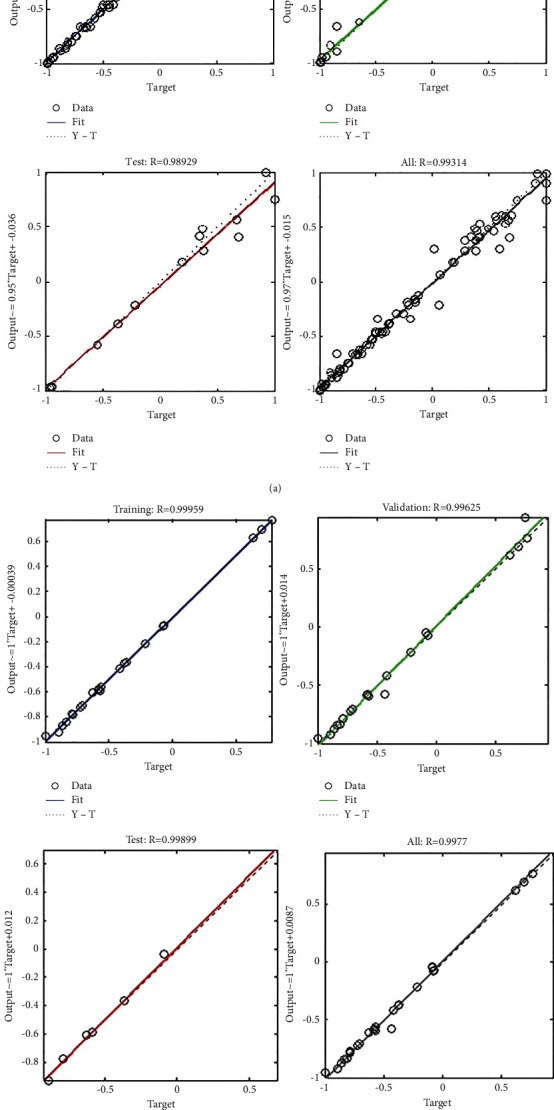
Linear regression diagram for training, evaluation, and testing of data. (a) FR method. (b) SVM method.

Figure 6(a) shows the results of machine learning based on the RF method and Figure 6(b) shows the results of machine learning based on the SVM method. According to the results of the two graphs presented in Figure 6, the data based on the three sets of input data are well trained and the presented results are more accurate. The linear regression diagram for training, evaluation, and testing of data obtained from machine learning by RF and SVM methods is also shown in Figure 7.

Based on the linear regression diagram of Figure 7, the correlation between trained and predicted data obtained from machine learning by the RF method (Figure 6(a)) is equal to 0.9931 and the correlation between trained and predicted data obtained from machine learning by the SVM method (Figure 7(b)) is equal to 0.9977. Therefore, in the continuation of the analysis, the learning machine method with SVM method has been used.

After training the data with RF and SVM machine learning methods, the SVM learning machine is selected because of its high correlation. To optimize the stock portfolio based on the purchase signal from Trading Filter Rule, Markov Model Filter, and Predict Model Filter, among 85 selected active stock exchange companies. Therefore, NSGA II and MOGWO algorithms have been used to optimize the stock portfolio based on the objectives of maximizing stock portfolio returns and minimizing stock portfolio risk in the Markowitz mean-variance model. Therefore, 7 different scenarios have been considered to investigate the solution methods. In scenarios 1 to 3, the stock buying signal of Tehran Stock Exchange companies is applied based on the performance of each of the filters, namely, Trading Rule Filter, Markov Model Filter and Predict Model Filter. In the fourth scenario, the buy signal is based on the combination of Trading Filter Rule, Markov Model Filter; in the fifth scenario, the purchase signal is based on the combination of Trading Filter Rule, Predict Model Filter, and in the sixth scenario, the purchase signal is based on the combination of Predict Model Rule, Markov Model Filter. Finally, in the seventh scenario, the purchase signal is examined based on the simultaneous application of all three filters.

**Figure 8:**
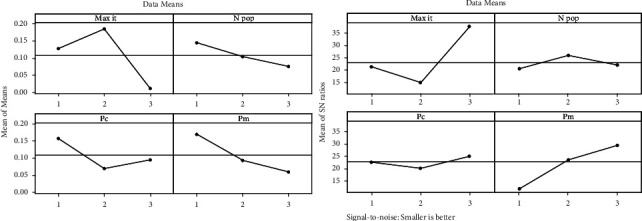
Average charts of *S*/*N* ratio and average in NSGA II algorithm.

**Figure 9:**
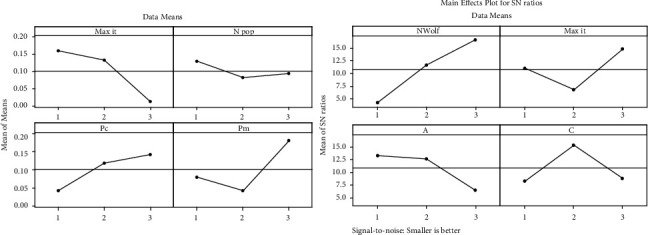
Mean charts of *S*/*N* ratio and average in MOGWO algorithm.

In each scenario, after presenting the stock purchase signals of the company in the coming days, NSGA II and MOGWO algorithms will be used to optimize the stock portfolio between 2016 and 2020. Therefore, in order to increase the efficiency of algorithms in maximizing stock portfolio returns and minimizing stock portfolio risk, the initial parameters of both algorithms should be adjusted by the Taguchi method. Therefore, 9 experiments are designed by the Taguchi method and the proposed algorithms are performed based on the levels presented in Table 2. The mean and mean *S*/*N* ratio graphs for selecting the optimal level of meta-heuristic algorithm parameters are shown in Figures 8 and 9.

According to Figures 7 and 9, it can be stated that the highest point in the average diagram of the *S*/*N* ratio is the selection of the desired level for setting the parameter of the meta-heuristic algorithm. Therefore, in order to increase the efficiency of the algorithms in optimizing the Markowitz mean-variance portfolio, in the NSGA II algorithm, the *N*pop parameter is 200, the Max *it* parameter is 200, and the *P*_*c* and *P*_*m* parameters are 0.7, and in the MOGWO algorithm, the *N* Wolf parameter. 300, Max *it* parameter 200 and parameters *A* and *C* are assigned values 1 and 2, respectively.

After setting the parameter of meta-heuristic algorithms, Table 3 shows the average stock portfolio return as well as the average stock portfolio risk obtained from different solution methods in 7 scenarios between 2016 and 2020.

**Figure 10:**
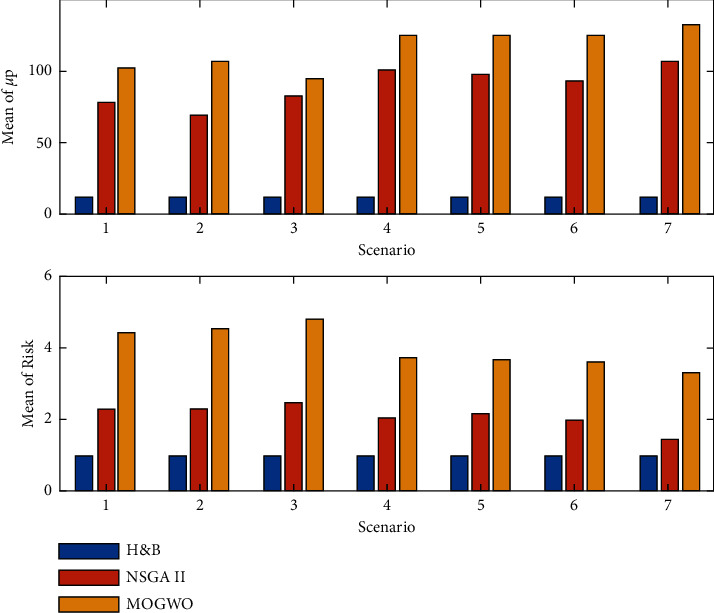
Bar chart comparing stock portfolio returns and investment risk in different scenarios.

According to the results obtained from Table 3, it can be seen that the MOGWO algorithm has a higher return than the NSGA II algorithm and the hold-and-buy (H&B) investment method. Also, the risk of investing in this algorithm in all scenarios is higher than the investment method with NSGA II and H&B methods. It is also observed by comparing stock portfolio returns and stock portfolio risk, if filters are used individually, stock portfolio returns are lower and investment risk is increased due to the possibility of a wrong buy signal. After training the data with RF and SVM machine learning methods, the SVM learning machine is selected because of its high correlation. To optimize the stock portfolio based on the purchase signal from Trading Filter Rule, Markov Model Filter and Predict Model Filter, among 85 selected active stock exchange companies. Thus, if all three filters mentioned in the literature are used, the average return of the portfolio by H&B method is 13.08% with a risk of 0.946%, while the use of meta-innovative algorithms as well as the use of all three filters in providing a buy signal has led to an average stock portfolio yield of 107.73% for the NSGA II algorithm and a portfolio yield of 133.13% for the MOGWO algorithm. The algorithm of Figure 10 compares the changes in stock portfolio returns and stock portfolio risk in different scenarios.

Also, based on the following relationships, the rate of return on investment (ROI) can be calculated in each of the scenarios for different solution methods. The amount of return on investment can be calculated based on relationships (13) and (14) per day of investment.

(13) $$\begin{array}{c} \begin{array}{cc} {\text{ROI}}_{t}=\frac{\left(1+\mu_{t}\right)}{100}, & \forall \;t=1 \end{array}. \end{array}$$

(14) $$\begin{array}{c} \begin{array}{cc} {\text{ROI}}_{t}=\frac{{\text{ROI}}_{t-1}\left(1+\mu_{t}\right)}{100}, & \forall \;t>1 \end{array}. \end{array}$$

In equation (13), *t* = 1 shows the rate of return on investment for the first period and in equation (14) for other periods.  Table 4 shows the rate of return on investment in different scenarios and with different solution methods.

**Figure 11:**
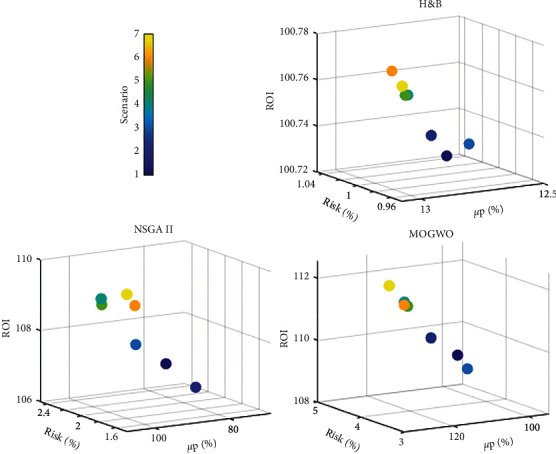
Comparison of average rate of return on capital in different scenarios.

Based on relations (7) and (8), it can be concluded that with the increase of the stock portfolio rate, the rate of return on capital increases. Therefore, the rate of return on capital obtained from the MOGWO algorithm is between 109.31 and 112.56 and the NSGA II algorithm is between 106.76 and 109.88. Also, if the filters are used simultaneously to provide a buy signal, the rate of return on investment will increase due to increased stock portfolio returns and reduced stock portfolio risk.  Figure 11 shows a comparison of the average portfolio return, portfolio risk, as well as the rate of return on investment in different scenarios for each method.

In the following, the return on investment portfolio between 2016 and 2020 in different scenarios with different solution methods is discussed. Therefore, Table 5 shows the average stock portfolio rate, stock portfolio risk, and return on investment in different years and in different scenarios.

**Figure 12:**
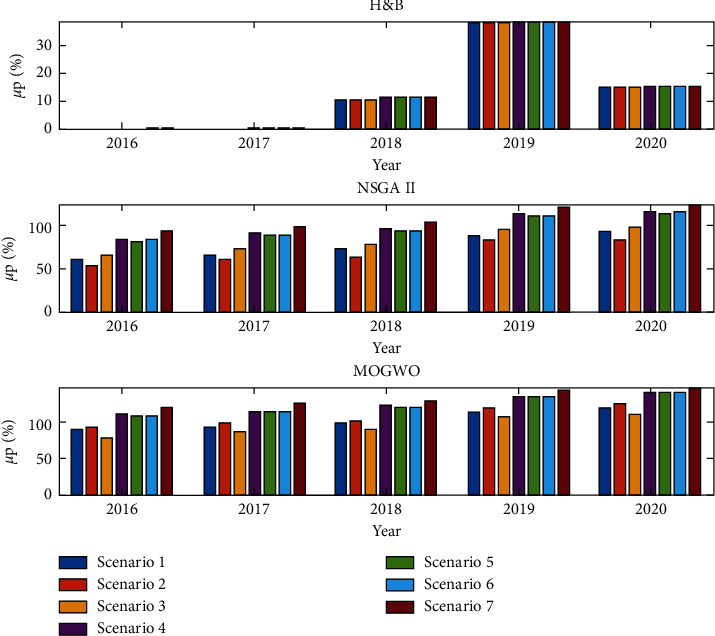
Comparison of stock portfolio rate in different scenarios and years (percentage).

**Figure 13:**
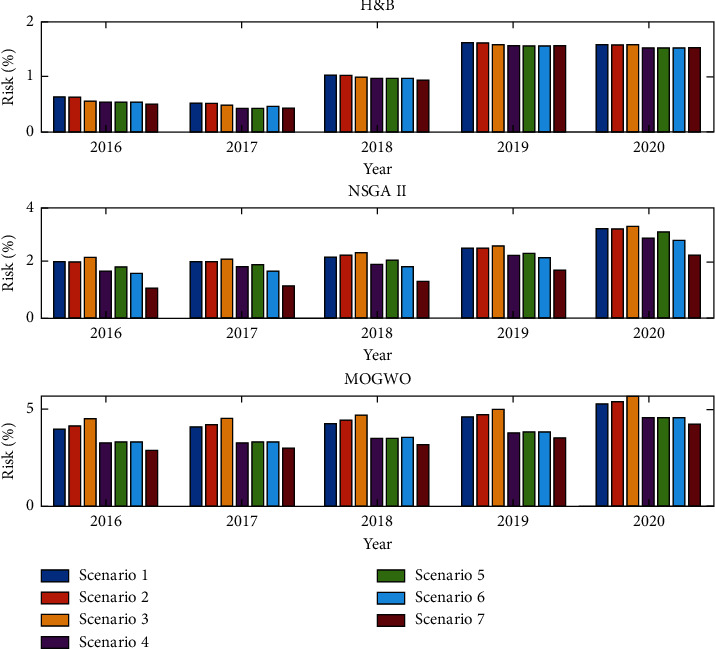
Comparison of stock portfolio risk in different scenarios and years (percentage).

According to the results of Table 5, the highest rate of return is related to the year 2020, in which the rate of return has increased due to the sharp growth of corporate stocks. The portfolio risk has also increased sharply this year. Figures 12 and 13 compare the stock portfolio rate of return and investment risk in each scenario with different solution methods.

Based on the obtained results, it can be seen that if all filters are used to provide a buy signal, the stock portfolio rate has been higher than other scenarios. However, in this method, the investment risk was lower than other methods due to providing a more accurate signal.  Table 6 compares the effect of different filtering methods in providing a buy signal on the stock portfolio rate and portfolio risk.

**Figure 14:**
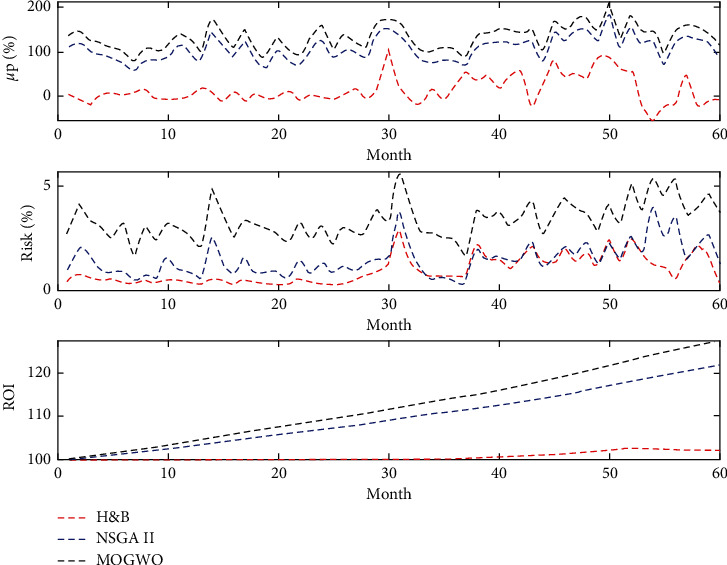
Comparison of trends in stock portfolio rate of return, investment risk, and return on investment in different months.

Due to the ideal of the hybrid filter method, Figure 14 compares the trend of changes in stock portfolio rate of return, investment risk, and return on investment in the 60 months leading to 2016 to 2020 with different solution algorithms.

After analyzing different solution methods in investing portfolio optimization, it was observed that the MOGWO algorithm has obtained the best average stock portfolio return in all years under test. The NSGA II algorithm, on the other hand, has a lower investment risk than the MOGWO algorithm.

Therefore, using the TOPSIS method and using various criteria, such as objective functions value, NPF, MSI, SM, MID, CPU-Time, it can be stated that the MOGWO algorithm with a utility weight of 0.652 has a higher efficiency than the NSGA II algorithm with a utility weight of 0.276 in determining the optimal stock portfolio.

## 6. Concluding Remarks and Future Suggestions

In this paper, a model for optimizing the stock portfolio of active companies listed on the Tehran Stock Exchange based on the Markowitz mean-variance model was presented. The main purpose of this model was to maximize the rate of return of the portfolio based on the forecasted price and reduce the risk of the portfolio simultaneously. Accordingly, 480 companies listed on the Tehran Stock Exchange between 2011 and 2020, along with all company stock information, have been extracted and filtered based on various inputs. To filter and select active stock exchange companies from 2011 to 2020, eight technical variables (final price, number of buyers, number of trades, trading volume, daily trading value, company daily value, *P*/*E* ratio, and number of shares of each company), seven fundamental variables (price of old design coin, new design coin, dollar, index, gold, oil, and euro), and time series of the final price of the last 10 days per share are considered and companies that have traded at least one day a month have been selected. Based on this, 85 companies active in the Tehran Stock Exchange have been identified and selected. After selecting 85 companies active in the Tehran Stock Exchange, to receive a buy signal from different combinations of three types of filters: “Optimization of trading rules based on technical analysis (Trading Rule Filter),” “Optimization of a strong trading system using Markov chain” (Markov Model Filter), and “Optimizing and Predicting Stock Returns Based on Predict Model Filter Methods.” In Trading Rule Filter, 6 indicators RSI, ROC, SMA, EMA, WMA, and MACD in six levels, namely, Very Very Weak (VVW), Very Weak (VW), Weak (W), Strong (S), Very Strong (VS), and very very strong (VVS) were used. Finally, in the Predict Model Filter, data were learned from machine learning methods based on two methods: random forest (FR) and support vector machine (SVM). In order to optimize the stock portfolio based on the Markowitz mean-variance model, two meta-innovative algorithms, MOGWO and NSGA II, as well as the hold and buy method (H&B) were used. Seven different scenarios were considered to investigate the methods. In scenarios 1 to 3, the stock buying signal of Tehran Stock Exchange companies is applied based on the performance of each of the filters, namely, Trading Rule Filter, Markov Model Filter, and Predict Model Filter. In the fourth scenario, the buy signal is based on the combination of Trading Filter Rule, Markov Model Filter; in the fifth scenario, the purchase signal is based on the combination of Trading Filter Rule, Predict Model Filter, and in the sixth scenario, the purchase signal is based on the combination of Predict Model Rule, Markov Model Filter. Finally, in the seventh scenario, the purchase signal is examined based on the simultaneous application of all three filters. The results of the analysis showed that the seventh scenario resulted in the highest stock portfolio return with the lowest investment risk. This is due to the provision of valid signals by three simultaneous filtering methods. In this scenario, the MOGWO algorithm obtained a 133.13% stock portfolio rate of return with a risk of 3.346%, while the stock portfolio rate of return on the NSGA II algorithm was 107.73, with a risk of 1.459%. Comparison of solution methods shows that the MOGWO algorithm has high efficiency in stock portfolio optimization.

By examining different solution methods in solving the stock portfolio optimization model, some suggestions can be made in developing and improving the quality of the article. Therefore, researchers can consider the uncertainty in stock portfolio investment and use uncertainty control methods such as robust programming. Also, in this paper, two meta-heuristic algorithms were used. Different and new meta-algorithms can be used to develop solution methods and compare them with each other. On the other hand, the existence of different methods of machine learning and their use is suggested.

## Figures and Tables

**Table 1:** Different examples of Markov processes for different levels.

	Countable state space	Continuous or general state space
Discrete-time	(Discrete-time) Markov chain on a countable or finite state space	Markov chain on a measurable state space (for example, Harris chain)
Continuous-time	Continuous-time Markov process or Markov jump process	Any continuous stochastic process with the Markov property (for example, the Wiener process)

**Table 2:** Parametric adjustment of meta-heuristic algorithms by Taguchi method.

Algorithm	Parameter	Level 1	Level 2	Level 3
NSGA II	*N*pop	100	150	200
	Max *it*	150	200	300
	*P*_*c*_	0.2	0.5	0.7
	*P*_*m*_	0.5	0.5	0.7

MOGWO	*N*wolf	100	150	200
	Max *it*	150	200	300
	*A*	1	2	3
	*C*	1	2	3

**Table 3:** The amount of stock portfolio returns and investment risk in different scenarios.

Scenario	Objective	H&B (%)	NSGA II (%)	MOGWO (%)
Scenario 1	Mean of *μp*	12.55	76.96	102.19
Scenario 2		12.62	69.01	107.65
Scenario 3		12.58	82.09	94.72
Scenario 4		12.94	100.52	124.89
Scenario 5		12.95	97.38	124.03
Scenario 6		12.98	93.38	125.51
Scenario 7		13.08	107.73	133.13

Scenario 1	Mean of risk	1.047	2.350	4.459
Scenario 2		1.045	2.350	4.596
Scenario 3		1.012	2.481	4.879
Scenario 4		0.980	2.077	3.713
Scenario 5		0.980	2.209	3.710
Scenario 6		0.987	1.995	3.662
Scenario 7		0.946	1.459	3.346

**Table 4:** Amount of rate of return on capital in different scenarios.

Scenario	H&B	NSGA II	MOGWO
Scenario 1	100.72	106.76	109.31
Scenario 2	100.73	105.99	109.88
Scenario 3	100.73	107.26	108.55
Scenario 4	100.76	109.14	111.73
Scenario 5	100.76	108.82	111.58
Scenario 6	100.77	108.92	111.67
Scenario 7	100.77	109.88	112.56

**Table 5:** Comparison of rate of return, stock portfolio risk and return on investment in different years (percentage).

Scenario	Year	*μp*			Risk			ROI		
		H&B	NSGA II	MOGWO	H&B	NSGA II	MOGWO	H&B	NSGA II	MOGWO
1	2016	−0.42	62.06	86.99	0.57	1.97	4.02	99.99	101.20	101.66
	2017	−0.26	67.61	93.27	0.48	1.99	4.07	100.01	103.62	105.05
	2018	10.27	72.46	97.73	1.01	2.16	4.28	100.18	106.38	108.84
	2019	38.14	89.25	114.22	1.62	2.45	4.63	101.07	109.30	112.88
	2020	14.88	93.42	118.75	1.57	3.17	5.31	102.40	113.34	118.12

2	2016	−0.32	54.43	92.79	0.57	1.93	4.16	99.99	101.06	101.78
	2017	−0.16	59.92	98.57	0.48	1.96	4.20	100.01	103.20	105.38
	2018	10.37	64.31	103.55	1.01	2.21	4.47	100.19	105.62	109.39
	2019	38.25	81.68	119.31	1.61	2.45	4.73	101.08	108.21	113.68
	2020	14.99	84.75	124.03	1.56	3.20	5.42	102.41	111.88	119.19

3	2016	−0.35	66.64	79.70	0.54	2.09	4.54	99.99	101.28	101.52
	2017	−0.19	73.18	85.76	0.44	2.12	4.50	100.01	103.90	104.64
	2018	10.33	77.80	90.53	0.97	2.32	4.69	100.18	106.86	108.10
	2019	38.21	94.38	106.41	1.58	2.62	4.99	101.07	110.02	111.82
	2020	14.94	98.47	111.24	1.53	3.26	5.68	102.41	114.28	116.68

4	2016	0.01	85.54	110.57	0.51	1.68	3.27	100.00	101.64	102.10
	2017	0.16	91.52	116.29	0.41	1.74	3.26	100.03	104.97	106.41
	2018	10.70	96.11	121.38	0.94	1.91	3.47	100.21	108.69	111.18
	2019	38.57	112.47	136.81	1.54	2.19	3.81	101.12	112.64	116.29
	2020	15.31	116.97	142.50	1.50	2.87	4.51	102.47	117.80	122.72

5	2016	0.01	82.27	109.16	0.51	1.82	3.31	100.00	101.58	102.08
	2017	0.17	88.39	114.81	0.42	1.82	3.32	100.03	104.78	106.33
	2018	10.70	93.24	119.70	0.94	2.08	3.52	100.22	108.38	111.03
	2019	38.57	109.18	135.55	1.54	2.29	3.86	101.12	112.19	116.06
	2020	15.31	113.82	140.98	1.50	3.03	4.54	102.47	117.19	122.43

6	2016	0.05	83.44	109.96	0.52	1.60	3.31	100.00	101.60	102.09
	2017	0.20	89.16	115.64	0.42	1.65	3.31	100.03	104.84	106.37
	2018	10.73	94.06	120.74	0.95	1.80	3.55	100.22	108.47	111.11
	2019	38.60	110.33	136.23	1.55	2.13	3.86	101.13	112.33	116.19
	2020	15.35	114.93	141.89	1.50	2.79	4.53	102.48	117.38	122.60

7	2016	0.15	92.74	118.21	0.47	1.06	2.90	100.00	101.77	102.26
	2017	0.30	98.70	124.72	0.38	1.11	3.00	100.04	105.38	106.87
	2018	10.83	103.42	128.55	0.90	1.28	3.16	100.23	109.40	111.99
	2019	38.70	119.56	144.62	1.51	1.61	3.48	101.14	113.68	117.45
	2020	15.45	124.23	149.55	1.46	2.25	4.20	102.49	119.20	124.28

**Table 6:** :Comparison of different filtering methods on stock portfolio optimization.

Scenario	Method	The best rank in earning the *μp*	The best rank in earning the risk
1	Trading filter rule	6	6
2	Markov model filter	5	7
3	Predict model filter	7	5
4	Trading filter rule + Markov model filter	3	4
5	Trading filter rule + predict model filter	4	3
6	Markov model filter + predict model filter	2	2
7	Trading filter rule + Markov model filter + predict model filter	1	1

## Data Availability

There is data in the article.
